# *ANRIL* Genetic Variants in Iranian Breast Cancer Patients

**DOI:** 10.22074/cellj.2017.4496

**Published:** 2017-05-17

**Authors:** Hamid Reza Khorshidi, Mohammad Taheri, Rezvan Noroozi, Shaghayegh Sarrafzadeh, Arezou Sayad, Soudeh Ghafouri-Fard

**Affiliations:** 1 Department of Surgery, Hamadan University of Medical Sciences, Hamadan, Iran; 2 Department of Medical Genetics, Faculty of Medicine, Shahid Beheshti University of Medical Sciences, Tehran, Iran

**Keywords:** *ANRIL*, Breast Cancer, Polymorphism

## Abstract

**Objective:**

The genetic variants of the long non-coding RNA *ANRIL* (an antisense noncoding RNA in the INK4 locus) as well as its expression have been shown to be associated with several human diseases including cancers. The aim of this study was to examine the association of *ANRIL* variants with breast cancer susceptibility in Iranian patients.

**Materials and Methods:**

In this case-control study, we genotyped rs1333045, rs4977574, rs1333048 and rs10757278 single nucleotide polymorphisms (SNPs) in 122 breast can- cer patients as well as in 200 normal age-matched subjects by tetra-primer amplification refractory mutation system polymerase chain reaction (T-ARMS-PCR).

**Results:**

The TT genotype at rs1333045 was significantly over-represented among pa- tients (P=0.038) but did not remain significant after multiple-testing correction. In addi- tion, among all observed haplotypes (with SNP order of rs1333045, rs1333048 rs4977574 and rs10757278), four haplotypes were shown to be associated with breast cancer risk. However, after multiple testing corrections, TCGA was the only haplotype which remained significant.

**Conclusion:**

These results suggest that breast cancer risk is significantly associated with *ANRIL* variants. Future work analyzing the expression of different associated *ANRIL* haplotypes would further shed light on the role of *ANRIL* in this disease.

## Introduction

**Fig.1:**
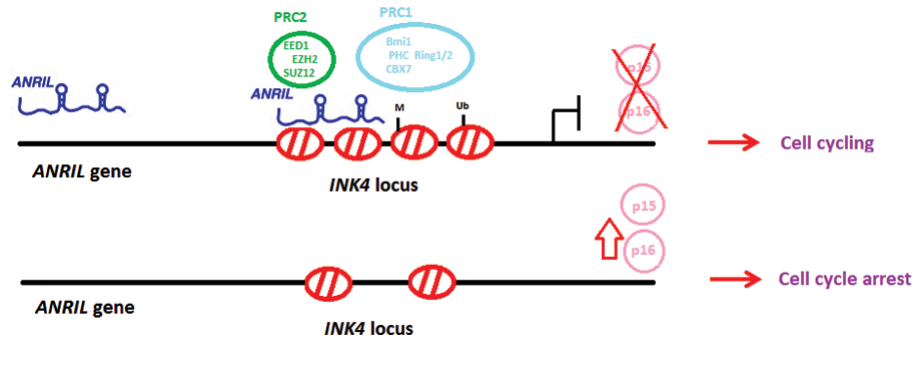
The location of *ANRIL* and its function in regulation of cell cycle.

Chromosome region 9p21 is a hotspot for disease-associated polymorphisms and encodes three tumor suppressors, namely p16^INK4a^, p14^ARF^ and p15^INK4b^, and the long non-coding RNA *ANRIL* (an antisense noncoding RNA in the INK4 locus) (1). This region has been shown to be altered in about one third of human tumors. *ANRIL* is a 3.8 kb-long non-coding RNA expressed on the reverse strand and has been shown to bind to and recruit PRC2 to repress the expression of p15INK4B (2). Figure 1 shows the genomic location of *ANRIL* and its function in regulation of cell cycle. *ANRIL* expression has been shown to be upregulated in breast cancer tissues with a significantly higher expression in triple-negative highly invasive cancers (3). Genome-wide association studies (GWAS) have identified *ANRIL* as a risk locus for numerous cancers such as breast cancer (4). This susceptibility may be explained by individual, tightly linked single nucleotide polymorphisms (SNPs) in the *ANRIL* locus; changing expression of *ANRIL* spliced transcripts and consequently influencing cellular proliferation pathways (5). *ANRIL* expression has been shown to be upregulated after DNA damage by the transcription factor E2F1. This suggests that *ANRIL* is involved in an ATM-dependent DNA damage response. In addition, increased levels of *ANRIL* inhibit the expression of p16^INK4a^, p14^ARF^ and p15^INK4b^ at the late-stage of DNA damage response (6).

Cardiovascular disorders are the most investigated human disorders which have been associated with *ANRIL* variants. For instance, rs4977574 has been shown to be strongly associated with the risk of coronary artery disease (7). On the other hand, rs11515 has been shown to be over-represented among breast cancer patients and has been associated with aggressive breast tumors, and higher *ANRIL* and lower p16^INK4a^ expression (1). Among other genetic variants within this gene, rs10757278 has been shown to increase the expression of the *ANRIL* variant EU741058 which contains exons 1-5 of the long transcript (8). Additionally, rs10757278 has been shown to modulate the *ANRIL* binding site for the transcription factor STAT1, which in turn regulates *ANRIL* expression (9). Considering the role of STAT1 in shaping an immunosuppressive tumor microenvironment in breast cancer cells (10), disruption of *ANRIL* binding site for STAT1 by the rs10757278 allele may be involved in breast cancer pathogenesis. Moreover, rs1333045 is an artery disease susceptibility SNP located in a conserved region in *ANRIL* which has been shown to have an enhancer activity in a reporter gene experiment (11). The other SNP within this gene, rs1333048, has been associated with the level of high sensitive C-reactive protein (hsCRP) which is a marker for systemic inflammation (12). Since recent studies have suggested an association between pre-diagnostic hsCRP and breast cancer risk as well as overall mortality (13), this variant might be associated with breast cancer susceptibility. 

The role of *ANRIL* in breast cancer pathogenesis and risk has been assessed in both expression and GWA studies; however, data regarding the role of specific SNPs within this gene in breast cancer susceptibility are scarce. Consequently, to find the association of *ANRIL* variants with breast cancer susceptibility in Iranian patients, we genotyped and examined the association of rs1333045, rs4977574, rs1333048 and rs10757278 according to their significance in the regulation of *ANRIL* expression and their participation in breast cancer-related pathways. 

## Materials and Methods

This case-control study was approved by the Ethical Committee of Hamadan University Hospital where 122 unrelated breast cancer patients, as well as 200 normal age-matched females from a routine health survey, were recruited during 2015 (IR.UMSHA.REC.1395.208). Informed consent was obtained from all participants. Clinical and pathological data of patients were collected. Diagnosis of breast cancer was confirmed by a pathologic study. 

### Single nucleotide polymorphism genotyping

Genomic DNA was extracted from blood samples of the patients and normal subjects using the standard salting out method. SNPs rs1333045, rs4977574, rs1333048 and rs10757278 were genotyped by tetra-primer amplification refractory mutation system PCR (T-ARMS-PCR) (14). PCR was performed in 25 µL total volume using Taq (2x) red master mix (Ampliqon, Denmark) and 0.5 µL of each forward and reverse primer (10 pmol) in a FlexCycler (Analytik Jena, Germany). The cycling conditions were an initial denaturation at 94˚C for 4 minutes, followed by 35 cycles of 94˚C for 45 seconds, annealing temperature for 45 seconds and 72˚C for 55 seconds, with a final extension of 72˚C for 5 minutes. Specific annealing temperatures were 45˚C for rs1333048, 53˚C for rs4977574, 52˚C for rs1333045 and 54˚C for rs10757278. The primers were designed by PRIMER1 (http://cedar. genetics.soton.ac.uk/public_html/primer1.html.) and are listed in Table 1. 

### Statistical analysis

The genotype and allele frequencies were calculated by direct counts. Deviation from the Hardy-Weinberg equilibrium was assessed using the Chi-square test. Pearson Chi-square test was utilized for comparing genotype and allele frequencies between the breast cancer patients and the control group using SPSS 16.0 (SPSS Inc., Chicago, IL, USA). Odds ratio (OR) and 95% confidence intervals (CI) were also calculated. These analyses were implemented. Haplotype frequencies for *ANRIL* were calculated using the SNPStats (http://bioinfo.iconcologia.net/SNPstats) based on the expectation- maximization algorithm (15). Pairwise linkage disequilibrium (LD) was assessed by calculating D' and squared correlation (r^2^) in Haploview (https:// www.broadinstitute.org/haploview/haploview) (16). D' was determined as the ratio of the unstandardized D to its maximal/minimal value. To avoid false positive results, permutation testing was performed (n=10,000) for multiple testing correction of the haplotype analysis. Differences were regarded as significant when P<0.05. 

## Results

Comparison of age between cases and controls showed no significant difference (mean age of patients: 38.9 ± 2.1 and mean age of healthy controls: 39.1 ± 1.8). The frequencies of all genotypes in both patients and control groups did not significantly deviate from Hardy-Weinberg equilibrium (P>0.05). The allele and genotype frequencies of the SNPs and the association results are shown in Table 2. Among all genotypes, only the TT genotype at rs1333045 was significantly more prevalent among patients (P=0.038), however, it did not remain significant after multiple-testing correction.

**Table 1:** Sequence of primers


Primer position	Primer sequence	PCR product size (bp)
rs1333045		

Forward inner primer (C allele)	CGAAGAGCAATAATATATAGTACACTGGGC	for C allele: 200
Reverse inner primer (T allele)	TTAATGAATGCTTACTAGATGCCTGA	for T allele: 298
Forward outer primer (5´-3´)	TGAAACTTCTTATTTAGTGGTGCATACC	by outer primers: 442
Reverse outer primer (5´-3´)	GCAGTTCAAAGGAAGTACCATAAAAAG	
**rs4977574**		
Forward inner primer (G allele)	TTGAGGGTACATCAAAAGCATTCTATATCG	for G allele: 226
Reverse inner primer (A allele)	TTTATTAGAGTGACTTGAACATCCCGT	for A allele: 166
Forward outer primer (5´-3´)	CACCATTCTTTCTGAAACAACAGGATAT	by outer primers: 335
Reverse outer primer (5´-3´)	AAGGCTCTGACATTTCTAACTCTCTGA	
**rs1333048**		
Forward inner primer (A allele)	TTAATGCTATTTTGAGGAGATGTCTA	for A allele: 185
Reverse inner primer (C allele)	TTTTATCAATATTTCAATAATTCGACACTG	for C allele: 253
Forward outer primer (5´-3´)	TTGCCTGATTACCAATTTTATATGTTA	by outer primers: 382
Reverse outer primer (5´-3´)	TCAACTGATGATGATATGGTTAGTATG	
**rs10757278 **		
Forward inner primer (A allele)	AAGTCAGGGTGTGGTCATTACGGGAA	for A allele: 263
Reverse inner primer (G allele)	CTCAGTCTTGATTCTGCATCGCTTCC	for G allele: 234
Forward outer primer (5´-3´)	GGGCATTAAGAAAtGGATGGGTAGACAAAA	by outer primers: 443
Reverse outer primer (5´-3´)	GCTGTTCTCAATTAGCCAGGACTACCTCT	


PCR; Polymerase chain reaction.

**Table 2:** Allele and genotype frequencies of *ANRIL* SNPs in the case and control groups


SNP	Model		Number (%)		Cancer vs. control	
			Cancer (%)	Control (%)	OR	P value

rs1333045	Allele	T vs. C	130 (53)	186 (46)	1.31 (0.95-1.80)	0.09
			114 (47)	214 (54)		
	Co-dominant	TT vs. CC	39 (32)	43 (21.5)	0.60 (0.32-1.11)	0.11
		CT vs. CC	52 (42.6)	100 (50)	1.05 (0.60-1.81)	
	Dominant	TT+CT vs. CC	91 (74.6)	143 (71.5)	0.85 (0.51-1.42)	0.54
			31 (25.4)	57 (28.5)		
	Recessive	TT vs. CT+CC	39 (32)	43 (21.5)	0.58 (0.35-0.97)	0.038
			83 (68)	157 (78.5)		
	Over dominant	TT+CC vs. CT	70 (57.4)	100 (50)	1.35 (0.86-2.12)	0.2
			52 (42.6)	100 (50)		
rs1333048	Allele	C vs. A	115 (47)	201 (50)	0.88 (0.64-1.21)	0.44
			129 (53)	199 (50)		
	Co-dominant	CC vs. AA	32 (26.2)	52 (26)	1.24 (0.68-2.28)	0.39
		CA vs. AA	51 (41.8)	97 (48.5)	1.45 (0.85-2.49)	
	Dominant	CC+CA vs. AA	83 (68)	149 (74.5)	1.37 (0.84-2.25)	0.21
			39 (32)	51 (25.5)		
	Recessive	CC vs. CA+AA	32 (26.2)	52 (26)	0.99 (0.59-1.65)	0.96
			90 (73.8)	148 (74)		
	Over dominant	AA+CC vs. CA	71 (58.2)	103 (51.5)	1.31 (0.83-2.06)	0.24
			51 (41.8)	97 (48.5)		
rs4977574	Allele	A vs. G	78 (32)	145 (36)	0.83 (0.59-1.16)	0.27
			166 (68)	255 (64)		
	Co-dominant	AA vs. GG	17 (13.9)	26 (13)	1.15 (0.57-2.31)	0.17
		GA vs. GG	44 (36.1)	93 (46.5)	1.59 (0.98-2.60)	
	Dominant	GA+AA vs. GG	61 (50)	119 (59.5)	1.47 (0.93-2.31)	0.096
			61 (50)	81 (40.5)		
	Recessive	AA vs. GA+GG	17 (13.9)	26 (13)	0.92 (0.48-1.78)	0.81
			105 (86.1)	174 (87)		
	Over dominant	GG+AA vs. GA	78 (63.9)	107 (53.5)	1.54 (0.97-2.45)	0.065
			44 (36.1)	93 (46.5)		
rs10757278	Allele	A vs. G	106 (43)	152 (38)	1.25 (0.91-1.73)	0.17
			138 (57)	248 (62)		
	Co-dominant	AA vs. GG	22 (18)	26 (13)	0.61 (0.30-1.21)	0.36
		GA vs. GG	62 (50.8)	100 (50)	0.83 (0.50-1.37)	
	Dominant	GA+AA vs.GG	84 (68.8)	126 (63)	0.77 (0.48-1.24)	0.28
			38 (31.1)	74 (37)		
	Recessive	AA vs.GA+GG	22 (18)	26 (13)	0.68 (0.37-1.26)	0.22
			100 (82)	174 (87)		
	Over dominant	GG+AA vs.GA	60 (49.2)	100 (50)	0.97 (0.62-1.52)	0.89
			62 (50.8)	100 (50)		


SNPs; Single nucleotide polymorphisms and OR; Odds ratio.

**Fig.2:**
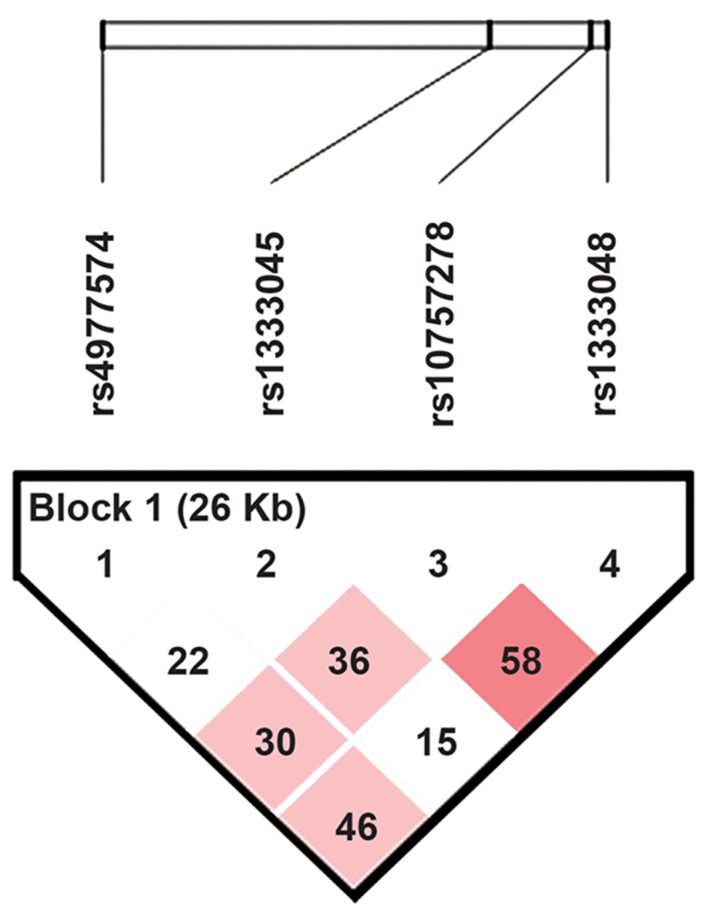
Linkage disequilibrium (LD) plot based on the four SNPs typed. The values in each cell represent D′ values.

Haplotype analysis was undertaken and distribution of haplotype frequencies in both groups was obtained (Table 3). Haplotype frequencies and the LD pattern (based on Dˊ) are shown in Table 3 and Figure 2 respectively. No significant LD was observed among the four SNPs (Dˊ<0.6). Among all observed haplotypes (with SNP order of rs1333045, rs1333048 rs4977574 and rs10757278), four haplotypes were shown to be associated with breast cancer risk. However, after multiple testing corrections, TCGA was the only haplotype which remained significant. Interestingly, this haplotype has the derived allele at SNPs rs1333045 and rs10757278, of which the former showed a hint of association with its homozygote form (TT).

**Table 3:** Haplotype frequencies of *ANRIL* SNPs in the case and control groups


rs1333045	rs1333048	rs4977574	rs10757278	Totalfrequency	Frequency in cancer group	Frequency in control group	OR (95% CI)	P value	Corrected P value

C	C	G	G	0.245	0.159	0.298	1.00	-	-
T	A	A	A	0.118	0.081	0.135	0.73 (0.35 - 1.53)	0.4	1
T	A	G	A	0.101	0.113	0.095	0.55 (0.27 - 1.11)	0.097	0.175
T	C	G	G	0.100	0.109	0.089	0.59 (0.27 - 1.28)	0.18	1
C	A	G	G	0.073	0.106	0.059	0.39 (0.17 - 0.88)	0.024	0.125
C	A	A	A	0.066	0.047	0.075	1.25 (0.42 - 3.77)	0.69	0.812
T	A	G	G	0.042	0.045	0.038	0.63 (0.24 - 1.64)	0.34	1
C	A	A	G	0.039	0.068	0.019	0.27 (0.09 - 0.84)	0.024	0.687
T	C	A	G	0.038	0.032	0.043	0.88 (0.28 - 2.73)	0.82	1
T	A	A	G	0.035	0.032	0.046	0.82 (0.25 - 2.63)	0.74	1
C	A	G	A	0.035	0.037	0.031	0.54 (0.17 - 1.67)	0.29	1
T	C	G	A	0.032	0.075	0.008	0.07 (0.01 - 0.39)	<10^-4^	0.002
C	C	A	G	0.026	0.014	0.028	1.31 (0.24 - 7.17)	0.76	0.812
C	C	G	A	0.025	0.036	0.019	0.43 (0.09 - 2.12)	0.3	1
T	C	A	A	0.023	0.045	0.012	0.23 (0.06 - 0.92)	0.039	0.687


## Discussion

*ANRIL* identification has accentuated the underrated role of genes encoding long noncoding RNA in pathogenesis of human disorders including cancers (5). Long noncoding RNA have been shown to have a regulatory role in telomere biology, chromatin dynamics, gene modulation and genome structural organization (17,18). Such a vast range of function suggests that they may also be involved in tumorigenesis processes. The role of *ANRIL* in regulation of DNA damage response makes it likely for it to have a critical role in breast cancer pathogenesis given that BRCA1, the most well-known breast cancer susceptibility gene mainly participates in DNA damage-induced cell cycle checkpoint activation and DNA repair (19). Other suggested roles for *ANRIL* include increasing cell proliferation and decreasing apoptosis (20) in addition to participation in inflammatory response; also emphasize its role in tumorigenesis (21). Many disease-associated SNPs identified by GWAS have been shown to be located in noncoding genomic regions which may contain long noncoding RNA such as *ANRIL*. Although in the current study, no genotype was strongly associated with breast cancer, one haplotypes was associated with breast cancer susceptibility in this population. Apart from rs11515 which has been assessed in breast cancer patients (1), the other three SNPs within this gene have not been genotyped in breast cancer patients. So the present study is among the first studies which have genotyped multiple SNPs within the *ANRIL* locus in these patients. The selected Considering the numerous splicing variants of *ANRIL* and the tissue specificity of some of the splicing variants (22), its physiological significance may be tissue- specific and so would be the effects of each SNP on its splicing variants. Haplotypes may be in closer linkage disequilibrium with a causal variant than any single SNP assessed and is thus more likely to show association. Furthermore, haplotypes may themselves be the causative variants of interest (23). Accordingly, our study also shows that haplotype analysis is more valuable than single SNP analyses. 

In addition, since the *ANRIL* genomic region encompasses various risk-associated SNPs (24), expression of *ANRIL* might be influenced by many of these genetic variants in linkage disequilibrium. Such deregulation of *ANRIL* by disease associated polymorphisms may change the expression level of p15^INK4B^ and/ or other target genes (2). Considering the role of p15^INK4B^ as a cyclin-dependent kinase inhibitor which prevents the activation of cyclin dependent kinases by cyclin D and functions as a cell growth regulator that inhibits cell cycle G1 progression (25), any change in its expression may have significant implications in tumorigenesis. 

## Conclusion

We show that *ANRIL* is associated with breast cancer susceptibility at the haplotype level. 

Further work comparing expression of *ANRIL* and its putative target genes based on different *ANRIL* haplotypes is necessary. 
